# Hydration and Properties of Cement in the Belite-Ye′elimite-Ternesite System

**DOI:** 10.3390/ma15082792

**Published:** 2022-04-11

**Authors:** Guoling Wang, Xiaofei Huang, Yufeng Wu, Qian Zhang, Suhua Ma, Weifeng Li

**Affiliations:** College of Materials Science and Engineering, Nanjing Tech University, Nanjing 211816, China; 201961103078@njtech.edu.cn (G.W.); 201961203147@njtech.edu.cn (X.H.); 202161203263@njtech.edu.cn (Y.W.); 202161203220@njtech.edu.cn (Q.Z.)

**Keywords:** belite-ye′elimite-ternesite, hydration, compressive strength

## Abstract

Energy consumption and carbon emissions are lower in the production of belite-ye′elimite-ternesite (C_2_S-C_4_A_3_$-C_5_S_2_$, BYT) clinker than Portland cement (PC) clinker. BYT cement can combine the early strength of CSA cements and the durability of belite cements. X-ray diffraction, mercury intrusion porosimetry, isothermal conduction calorimetry and scanning electron microscope were conducted to investigate the hydration process of BYT cement. The hydration products of BYT cement include mainly ettringite, strätlingite and some amorphous AH_3_ (aluminum hydroxide). Ternesite did prove an early reactivity in BYT cement. The reaction of ternesite with AH_3_ occurs on the surface of ternesite. Ternesite delays the second heat flow peak of ye′elimite. The strength of BYT cement containing 10% ternesite in the prepared clinker exceeds that of other cement at all ages.

## 1. Introduction

The cement industry is facing increasing pressure due to the depletion of natural fuel resources, shortage of raw materials, continuously increasing cement demand and concerns about climate-linked environments [1]. Calcium sulfoaluminate (CSA) clinker has a lower calcium content and therefore releases less CO_2_ from the decomposition of calcium carbonate during the clinker calcination process than that of PC clinkers [2,3]. CSA clinker can be produced within a temperature range of 1250–1350 °C, which is 100–150 °C lower than that in the production process of PC clinker; therefore, fossil fuel consumption is decreased [4,5]. In addition, power consumption is reduced significantly to produce CSA cement, which has a good characteristic of grindability [6]. CSA cement has some advantages, such as high early strength, rapid setting, sulfate resistance and shrinkage compensating properties [7,8]. Hence, CSA cement is considered a promising alternative to PC cement. The main phases in the CSA clinker include belite, ye’elimite and smaller amounts of ferrite and aluminate. The fast hydration of ye′elimite results in higher early strength of CSA cement, and belite provides power for improving the later strength [9,10]. In the past, ternesite was considered a hydrated inert mineral. However, Bullerjahn [6,11] et al. demonstrated that ternesite reacted already at early hydration ages. In later work, they revealed that the reaction was linked to the presence by AH_3_. Ternesite bridges the reactivity gap between the rapid aluminate reaction and the late strength contribution of belite [12,13]. Belite-ye′elimite-ternesite (BYT) cement combines the early strength of CSA cements and the durability of belite cements [13]. The contributions of major clinker minerals to the evolution of the early performance and properties of BYT cement are in the following order: ye′elimite >> ternesite > belite [11].

Ye′elimite is generally considered to react ideally in the presence of water to form monosulphate (AFm) and microcrystalline gibbsite (AH_3_) according to reaction Equation (1) [14,15]. Bullerjahn and co-authors demonstrated that, indeed, the “ideal” reaction does not occur, which is linked to the formation of a metastable phase assemblage composed of AH_3_ gel, calcium aluminate hydrate and metastable ettringite. The hydration products of cements dominated by ye′elimite could be adjusted by adding varying gypsum types and contents [16,17]. Ettringite and AH_3_ are preferentially formed when gypsum is present according to reaction Equation (2) [14,18]. When gypsum is depleted completely, the formation of AFm becomes the dominant reaction [19]. However, the hydration processes of ye′elimite-rich cements are much more complicated than these simplified hydration models. Metastable phases, such as C_4_AH_13_, C_2_AH_8_ and CAH_10_, have been reported in CSA cement systems with variable gypsum contents [20,21,22]. The addition of inert filler, such as quartz, further enhances the heterogeneous nucleation of ettringite, CAH_10_ and amorphous AH_3_ [14]. For the optimum setting time, strength development and volume stability, the content of gypsum in CSA cement is approximately 16–25 wt.%, which is much higher than that of PC [23].

(1) $$C_{4}A_{3}\$+18H\to C_{6}{A\$H}_{12}+2{\mathrm{AH}}_{3}$$

(2) $$C_{4}A_{3}\$+2{C\$H}_{2}+34H\to C_{6}{A\$}_{3}H_{32}+2{\mathrm{AH}}_{3}$$

The hydration behavior of belite in PC mainly occurs at later ages. The hydration products of belite are mainly amorphous calcium silicate hydrate (C-S-H) gel and portlandite (CH) (Equation (3)) [24,25]. The hydration process of belite changes due to the formation of AH_3_ from ye′elimite in the ye′elimite-rich cements [26]. The hydration product of belite is mainly strätlingite in the presence of AH_3_ (Equation (4)) [27].

(3) $$C_{2}S+\left(2-x+y\right)H\to C_{x}{\mathrm{SH}}_{y}+\left(2-x\right)\mathrm{CH}$$

(4) $$C_{2}S+{\mathrm{AH}}_{3}+5H\to C_{2}{\mathrm{ASH}}_{8}$$

The hydration of ternesite must be stimulated by AH_3_ in pores solution according to Equation (5) [28]. Montes et al. noted that all aluminates (C_3_A, C_12_A_7_, C_4_A_3_$ and CA) could activate the hydration of ternesite and that the activation degree of aluminate to ternesite is C_12_A_7_ ≈ CA > C_3_A >>>> C_4_A_3_$ [29,30]. The reactivity of ternesite also depends on the curing temperature and humidity, particle fineness and size distribution [31].

(5) $$C_{5}S_{2}\$+2{\mathrm{AH}}_{3}+10H\to 2C_{2}{\mathrm{ASH}}_{8}+2C\$$$

Bullerjahn believed that the hydration of ternesite could only occur when gypsum was completely consumed [11]. The amount of gypsum addition mainly depends on the content of ye′elimite.

This paper studies the hydration process of a BYT cementitious system in the presence of gypsum. It is necessary to incorporate a large amount of gypsum, which can control the type and quantity of hydration products, inhibit the formation of AFm and promote the formation of ettringite. The purpose of this study is to understand the hydration and performance of the BYT cementitious system, including the evolution of the phase combination with hydration time and the mechanical and microstructural properties. The system contains 5%, 10%, 15% 20% and 25% ternesite. The key effect and hydration activity of ternesite are discussed.

## 2. Experimental Section

### 2.1. Raw Materials and Sample Preparation

The raw materials used in the experiment include calcium carbonate (CaCO_3_), silicon dioxide (SiO_2_), gypsum (CaSO_4_·2H_2_O) and aluminum oxide (Al_2_O_3_), which were all analytical grade reagents. Raw materials were weighed accurately according to the stoichiometric C_2_S, C_4_A_3_$ and C_5_S_2_$. The designed phase composition of clinkers and amount of raw materials are shown in Table 1.

Clinker preparation: the raw materials were mixed in a planetary ball mill for 12 h to ensure raw mix homogeneity. A pressure of 20 MPa was applied to prepare 4 cm diameter cylindrical pellets. All pellets were dried at 110 °C. The dried pellets were calcined at 900 °C with a heating rate of 10 °C/min and held for 0.5 h to completely decompose the calcium carbonate. Then, BYT-1, BYT-2 and BYT-3 were heated to 1180 °C, and BYT-4 and BYT-5 were heated to 1210 °C for 1 h to obtain BYT clinkers. Each calcined sample was quenched by air flow.

Cement preparation: BYT cements were obtained by adding gypsum into BYT clinkers. The amount of gypsum added was determined according to Equation (6) [27,32].

(6) $$C_{G}=0.13\times M\times \frac{A_{C}}{\overset{\text{¯}}{S}}$$

C_G_ is the ratio of gypsum to clinker, A_C_ is the mass fraction of ye′elimite formed in clinker, $\bar{S}$ is the mass fraction of SO_3_ in gypsum, M is the gypsum coefficient (M = 2), and ye′elimite is hydrated together into ettringite. The content of clinker and gypsum in each group of cement is listed in Table 2.

Hydration sample preparation: BYT cement sample was mixed with water according to the mass ratio (*w*/*c*) of 0.5. The slurries were stored in a plastic centrifugal tube at a temperature of 20 ± 1 °C and 95% RH. Hydrated samples were cut into 3 mm discs with a precision cutting machine at different ages. The discs were immersed in anhydrous ethanol for one week to stop hydration. After stopping hydration, parts of the discs were ground into powder for X-ray diffraction (XRD) and thermogravimetric-differential scanning calorimetry (TG-DSC) analysis, and the other discs were used for mercury intrusion porosimetry (MIP) analysis.

### 2.2. Experiment Methods

#### 2.2.1. X-ray Fluorescence (XRF) Analysis

X-ray fluorescence (XRF) spectrometry was used to measure the bulk chemical composition. Measurements were performed using a Philips PW2400 XRF spectrometer (Philips, Amsterdam, The Netherlands), and the data were analyzed with the Uniquant^®^ software suite (Version: 8, Thermo Scientific, Waltham, MA, USA). The XRF data were used to calculate the mass attenuation coefficients (μ_m_) of the clinker samples used in the quantitative X-ray diffraction. Table 3 presents the chemical composition and μ_m_ value of the five clinkers.

#### 2.2.2. X-ray Diffraction Analysis

The XRD data were collected by a Miniflex600 X-ray diffractometer (Rigaku, Matsumoto, Japan) at 20 ± 1 °C. The diffractometer generator was fitted with a Cu Kα X-ray source (k = 1.54059 Å) operating at 40 kV and 15 mA. The range of 2 theta is 5° to 70° with a step size of approximately 0.01°. HighScore Plus software (version 5.1, Panalytical, Almelo, The Netherlands) was applied for the phase identification and quantification.

In order to obtain an absolute quantification of the crystalline phase content, the external standard approach was used. The Rietveld refinements were carried out based on the structure models shown in Table 4. The refined parameters included background coefficients, cell parameters, peak shape parameters and phase scales. The background was fitted graphically with the shifted Chebyshev polynomial using 10 coefficients. The peak shapes were fitted by a Gauss–Lorentz function. In this article, R-weighted pattern (Rwp) is less than 10. For major phases (more than 20 wt.%), the percent error ranges are less than 5%. The external standard approach was used to obtain an absolute quantification of the crystalline-phase content. The diffractometer constant G was determined by standard NIST Al_2_O_3_. This G factor was used to determine the mass concentration of each mineral phase of the BYT clinker and hydration products of the BYT cement through the XRD Rietveld quantification method using Equation (7):

(7) $$W_{\alpha }=S_{\alpha }\frac{\rho_{\alpha }V_{\alpha }^{2}\mu_{\alpha }}{G}$$

where $\rho_{\alpha }$ is the density of phase α, $V_{\alpha }$ is the unit-cell volume of phase α, $W_{\alpha }$ is the weight fraction of phase α, and μ_α_ is the mass attenuation coefficient of the quantified sample, which is provided in Table 3 and calculated by the content of each oxide in the sample [33].

#### 2.2.3. Initial Characterization of the Anhydrous Cements

**Figure 1:**
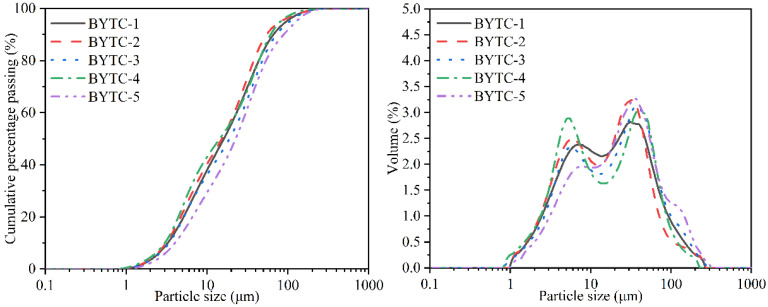
Particle size distribution of the BYT cement powder.

The textural properties are as important as the phase contents in the aspect of interpreting the reactivities. Therefore, a thorough textural characterization was carried out. The fired clinker was crushed and ground. Particle size of BYT-1~5 cement ranged from 0.89 μm to 271.4 μm, and the mean particle size (D_50_) was 16.96, 15.56, 20.17, 14.27, 28.53 μm, respectively (Figure 1). Powders were first dried at 110 °C for 1 h, and then, the fineness of five BYT cements was measured in a Blaine fineness apparatus (SBT-127) according to JB-T 8074-2008. Their Blaine specific surface area was about 420 ± 10 m^2^/kg.

#### 2.2.4. Isothermal Conduction Calorimetry

Isothermal conduction calorimetry (8-channel TAM AIR calorimeter, Thermometric AB, Stockholm, Sweden) was used to measure the hydration heat liberation of BYT clinkers during the first 72 h at a constant temperature of 20 °C. To measure the initial thermal response of the samples, an internal stirring method was used in this experiment. An amount of 4 g of BYT clinker was weighed into a flask, and 2 g of water was added to an injector, which was then placed in the measuring position of the instrument. Before testing, the samples rested for 45 min to stabilize their temperature and the water temperature.

#### 2.2.5. Thermogravimetric Analysis-Differential Scanning Calorimetry

To determine the cement hydration products, a TGA/DSC 1/1600LF synchronous thermal analyzer (Mettler toledo, New York, NY, USA) was used to perform a TG-DSC analysis of the hydration samples. Weight loss was measured by heating samples from room temperature to 1000 °C at a uniform heating rate of 10 °C/min in a nitrogen atmosphere.

#### 2.2.6. Electron Microscopy

Microstructure and morphology of pastes were identified using a Zeiss-Supra 55 Scanning Electron Microscopy (SEM) (Carl Zeiss, Oberkochen, Germany) with an acceleration voltage of 15 kV. The sample was sputtered with gold in the vacuum to improve electrical conductivity. The backscattered scanning electron (BSE) mode was chosen, and the accelerating voltage was 15 kV. The elemental composition of the pastes was determined by energy dispersive spectroscopy (EDS) using an X-max 150 X-ray energy spectrometer (Oxford instruments, London, UK).

#### 2.2.7. Mercury Intrusion Porosimetry

Porosity and pore size distribution were measured for the specimen fragments using mercury intrusion porosimetry (MIP, Quanta chrome PoreMaster 60 GT). A major advantage of MIP is that it has a wide range of pore size characteristics. With increasing pressure, the mercury breaks the interfacial tension and intrudes into the pores of cement paste. The applied pressure and the amount of mercury intrusion were used to determine the corresponding pore size and quantity. The pressure applied by the intrusion porosimeter ranged from 0 to 206 MPa, and the pore size in the range of 0.007 μm to 230 μm was measured.

#### 2.2.8. Compressive Strength

BYT cement was used to test the compressive strength with a *w*/*c* ratio of 0.5. The slurry was stabilized in a 20 × 20 × 20 mm test mold, and the slurry was cured in a curing box with a temperature of 20 ± 0.1 °C and RH 95% for 24 h. Then, the test block was placed in water at 20 ± 0.1 °C to continue the curing process. The compressive strength was tested at 1 d, 3 d, 7 d, 28 d, 56 d, 90 d and 180 d.

#### 2.2.9. The Degree of Hydration

The degree of hydration (DoH) was calculated from the result of XRD-Rietveld refinement, according to Equation (1) where the $w_{t0}(C_{2}S)$, $w_{t0}(C_{4}A_{3}\$)$, $w_{t0}(C_{5}S_{2}\$)$ represent the initial content of minerals phases in anhydrous BYT cement, and $w_{t}(C_{2}S)$, $w_{t}(C_{4}A_{3}\$)$, $w_{t}(C_{5}S_{2}\$)$ represent the content of minerals in hydrated pastes at t. The content of minerals was normalized by the weight loss obtained from TG-DSC.

$$\mathrm{DOH}=1-\frac{w_{t}(C_{2}S)+w_{t}(C_{4}A_{3}\$)+w_{t}(C_{5}S_{2}\$)}{w_{t0}(C_{2}S)+w_{t0}(C_{4}A_{3}\$)+w_{t0}(C_{5}S_{2}\$)}$$

## 3. Results and Discussion

### 3.1. Phase Composition of BYT Clinker

**Figure 2:**
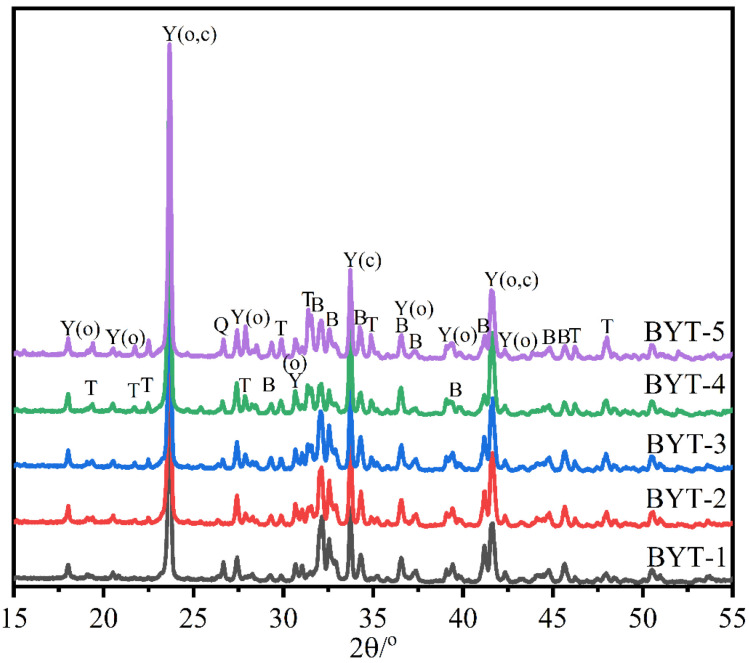
X-ray diffraction pattern of the BYT clinkers (B-C_2_S, Y-C_4_A_3_$, T-C_5_S_2_$, Q-SiO_2_).

The XRD patterns of the synthesized BYT clinkers are shown in Figure 2. BYT clinkers were synthesized at different temperatures, mainly because the content of ternesite was sensitive to temperature. The previous investigation claimed that the calcined temperature should be at 1180 °C for BYT clinkers with less than 15% ternesite and at 1210 °C for BYT clinkers with more than 15% ternesite. The XRD patterns mainly contain diffraction peaks of belite, ye′elimite, ternesite and quartz for all the clinkers. Two polymorphs of ye’elimite were formed, including orthorhombic and cubic crystal forms. The phase compositions quantified through Rietveld analysis are shown in Table 5. The results show that the three targeted phases (belite, ye′elimite and ternesite) formed adequately, and no free CaO was detected in the synthesized clinkers. The results indicated that these BYT clinkers were synthesized successfully.

### 3.2. Hydration Kinetics

**Figure 3:**
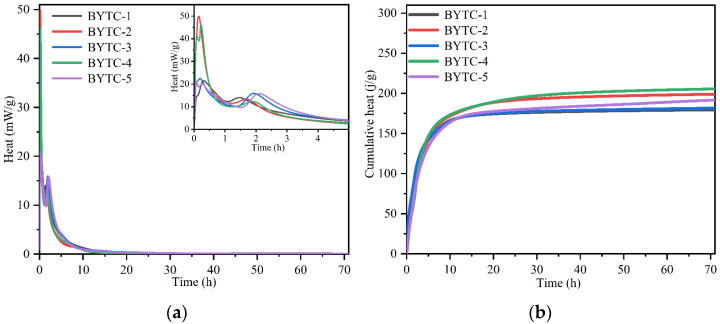
Isothermal calorimetry curves of per gram of cement (**a**) rate of hydration; (**b**) cumulative heat of hydration.

Figure 3 shows the heat evolution curves and cumulative heat of the BYT cements. Only two main peaks were observed in the curves of all BYT cements, and the induction period between the two exothermic peaks was very short. Previous studies have shown that the first exothermic peak occurs directly when the water is added and can be attributed to the solution heat and the rapid hydration of ye′elimite. The first peak is higher for the BYTC-2 and BYTC-4 in comparison with the other cements because the contents of ye′elimite are the highest. For the BYTC-1, BYTC-3 and BYTC-5, their first exothermic peaks are similar due to the same content of ye′elimite in these samples. It was reported that the second exothermic peaks occur after the massive dissolution of ye′elimite (+gypsum) and precipitation of ettringite (+AH_3_) [43]. The second peak of cement BYTC-1, BYTC-2, BYTC-3, BYTC-4 and BYTC-5 appeared at approximately 1.5 h, 1.7 h, 1.9 h, 1.9 h and 2.1 h, respectively. It seems that the time when the second exothermic peak appears is related to the content of ternesite. The higher the content of ternesite in the system, the later the second exothermic peak appears. It demonstrated that ternesite delays the second exothermic peak of ye′elimite. After 10 h hydration, the heat flow is close to zero for all the samples. It can be seen from the hydration heat release diagram that the accumulated heat release of BYTC-4 cement is the highest and can reach 205 J/g at approximately 30 h hydration, mainly because the content of ye′elimite in this cement is the highest. Through the hydration heat analysis of different BYT cement compositions, when the hydration process reached 30 h, the hydration curve basically remained stable, indicating that the hydration process entered a stable period and that hydration was completely controlled by the diffusion rate. 

### 3.3. Phase Composition of the Hydrated Cement Paste

**Figure 4:**
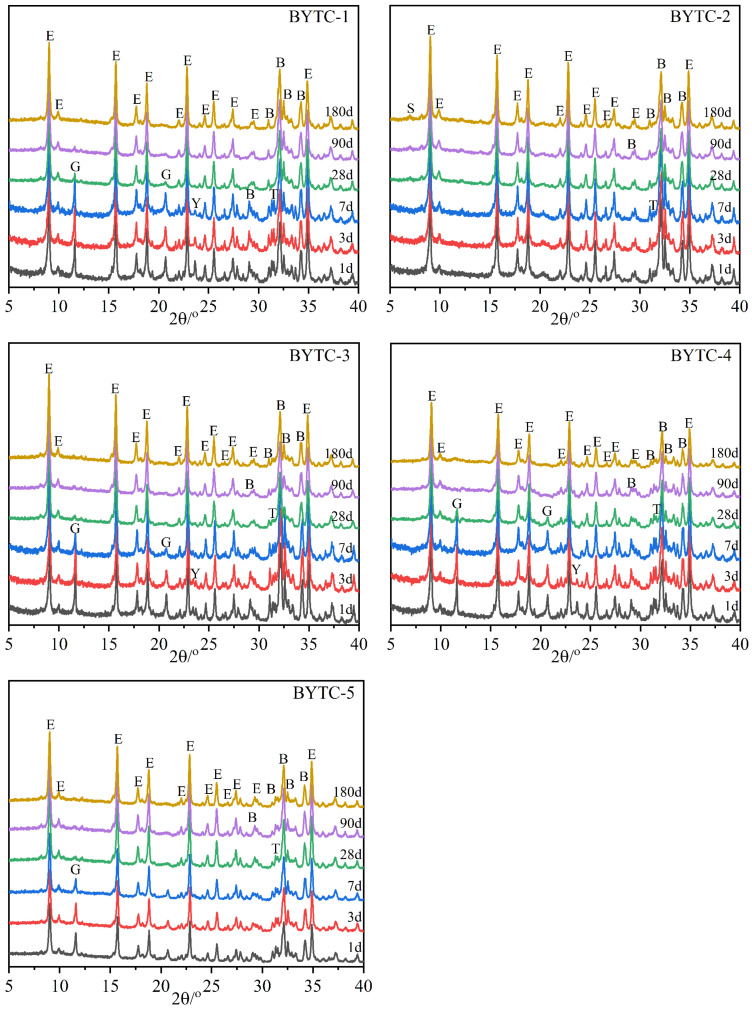
Diffractograms of hydrated pastes of BYT cement (S—strätlingite).

The XRD spectra of hydration of BYT cements at different ages are listed in Figure 4. The major diffraction peak varies within the range of 2 theta between 5° and 40°. The variation of phase compositions was similar with hydration age for all the samples. At the early hydration (from 1 d to 28 d), the phase assemblages included the remained phases (ye′elimite, belite); neither portlandite nor C-S-H was observed. The absence of AH_3_ in XRD patterns is related to its amorphous nature [44]. The diffraction peak of ye′elimite basically disappeared in the first 7 d of hydration, corresponding to the rapid weakening of the gypsum diffraction peak and the rapid strengthening of the ettringite diffraction peak. The strätlingite diffraction peak is observed only in the diffraction pattern of BYTC-2 cement, which is related to the rapid consumption of gypsum in the paste. The peak intensity of ternesite decreased in the first 28 d of hydration, but part of the peak remained. Gypsum from the hydration of ternesite was not found, probably because it was involved in the formation of ettringite. These results indicated that ternesite can be activated in the presence of ye′elimite.

**Figure 5:**
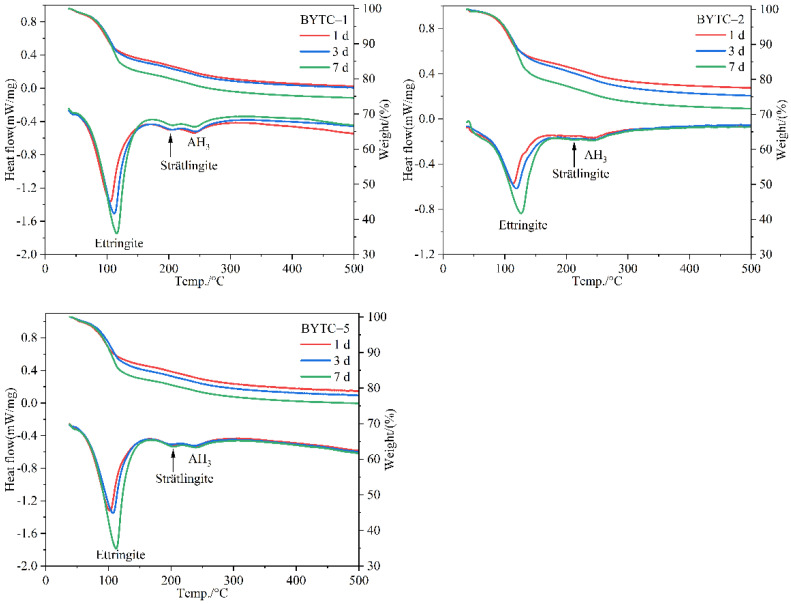
TG-DSC curves of BYT cements hydration for 1 d, 3 d and 7 d.

The main hydration products were further determined by TG-DSC analysis. TG-DSC curves of BYTC-2 cement with different hydration ages are shown in Figure 5. The two endothermic peaks of the hydrated sample are located at 120 °C and 250 °C. The presence of the first endothermic peak was due to the thermal decomposition of ettringite, leading to the greatest weight loss. Weight loss at around 200 °C was related to the decomposition of strätlingite. The third endothermic peak was due to the dehydration of AH_3_, which was formed during the hydration of ye′elimite and was partly consumed during the hydration of belite and ternesite. AH_3_ is not observed in the XRD patterns, which may be due to its lower weight percentage and poor crystallinity. The total weight loss percentages of the hydrated samples on 1 d, 3 d and 7 d were 22.71%, 25.07%, 28.76%, respectively.

### 3.4. The Hydration Degree of BYT Cement

**Figure 6:**
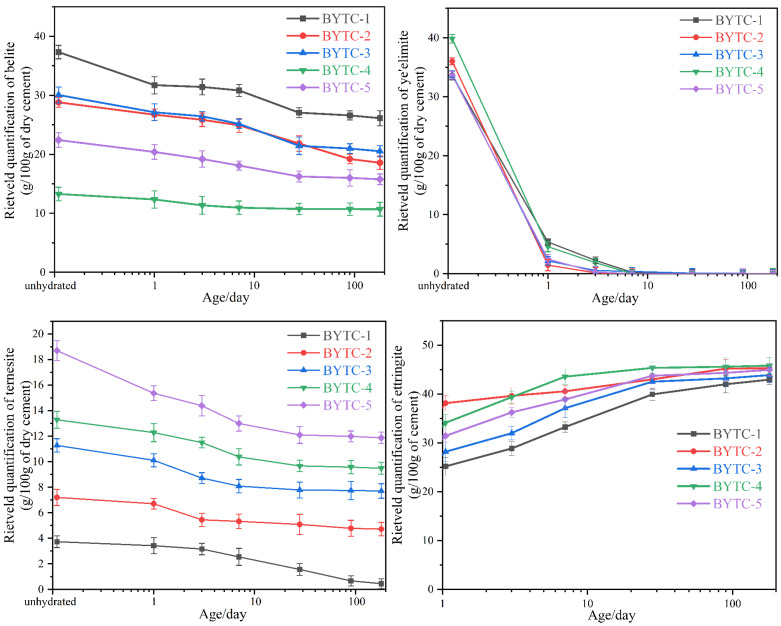
Evolution of belite, ye′elimite, ternesite and ettringite obtained by Rietveld refinement (measurement error is ±2%). Note: the *y*-axis scale is different.

The XRD Rietveld quantification method was used to determine the content of belite, ye′elimite and ternesite at different ages (Figure 6). The content of ettringite increases particularly rapidly in the first 7 d of hydration, which corresponds to the sharp decrease in the ye′elimite content. Afterward, the content of ettringite increases slowly, which is related to the crystallinity improvement. It should be noted that because ettringite (as the main hydration product) is combined with a large amount of water, the mass fraction of various phases can be diluted to varying degrees in different BYT cements and hydration ages. Therefore, three-mineral phase content is normalized by the anhydrous sample mass. The early hydration activity of belite was higher in BYT cement than in PC cement. The initial contents of belite in BYTC-2 and BYTC-3 were similar. After 28 d of hydration, the content of belite in BYTC-3 changed very slowly, while that in BYTC-2 slowed down after 90 d. The difference in the content of ternesite may contribute greatly to the activity of belite. Ye′elimite is almost completely consumed after 7 d of hydration, which corresponds to its high hydration activity. The content of ternesite decreased significantly in the first 7 d because the hydration activation of ternesite was stimulated in the presence of ye′elimite. The hydration activity of the three minerals is inconsistent in different design compositions, which also indicates that appropriate composition design may result in better synergy in the hydration process of the three minerals.

**Figure 7:**
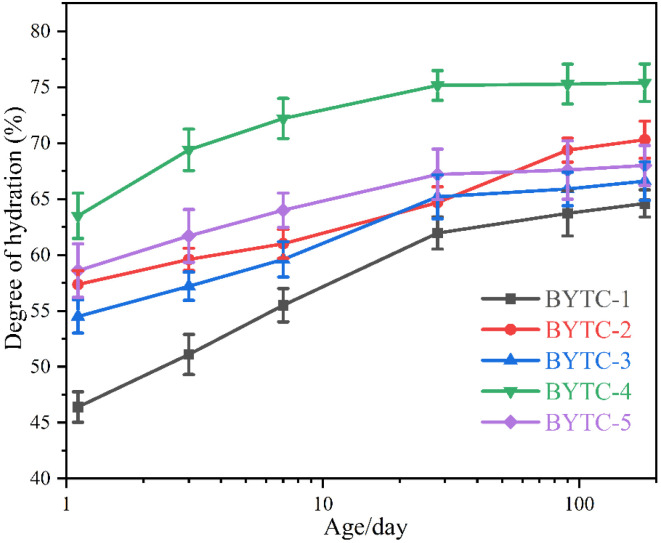
Hydration degree of BYT cement (measurement error is ±2%).

Figure 7 shows the hydration degree (DoH) of BYT cement at all hydration ages. The hydration degree of cement is mainly related to the content of ye′elimite. Due to the high hydration activity of ye′elimite, when the content of ye′elimite increases, the hydration degree of cement is higher. BYTC-4, which has the highest ye′elimite content (60 wt.% in clinker), has the highest hydration degree, followed by BYTC-2 (50 wt.% in clinker). It is noteworthy that while the ye′elimite contents in BYTC-1, BYTC-3 and BYTC-5 are comparable, the contents of belite and ternesite are different, and the hydration degree of samples with more ternesite is higher, which indicates that the hydration activity of ternesite is higher than that of belite.

### 3.5. Characterization of the Microstructure of the Hydrates

**Figure 8:**
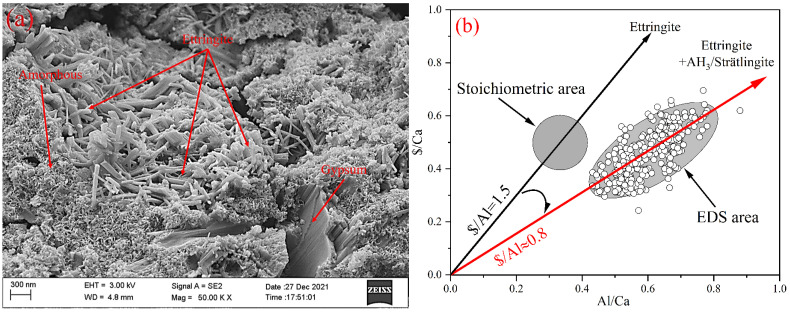
(**a**) SEM images of the hydration products of BYTC-2 formed after 7 d (on fractured surfaces); (**b**) Al/Ca and $/Ca molar ratios as determined by EDS analysis.

Figure 8 shows the typical microstructure of hardened BYT cement systems hydration for 7 d. Most areas of hardened slurry are morphologies as shown in Figure 8a. A great quantity of needle and columnar ettringite appeared in the hydrated sample, as well as a large amount of amorphous gels. Amorphous gels are attached to ettringite surfaces and interstices. Song [45] et al. proved that the amorphous gels were mainly composed of AH_3_ precipitated onto ettringite surface by EDS and TEM. Padilla-Encinas [46,47] et al. considered that amorphous gels and low crystallinity AH_3_, which found it easier to fill the pores between the ettringite needles, could improve the matrix properties. The results of EDS on ettringite surface show a higher content of aluminum than stoichiometric ettringite, which can be explained by the hydration equation of ye′elimite (Equation (2)). Compared with stoichiometric ettringite, the co-existence of ettringite and AH_3_ leads to a higher proportion of Al element. In addition, hydration product strätlingite of belite and ternesite also affects the ratio of the elements.

**Figure 9:**
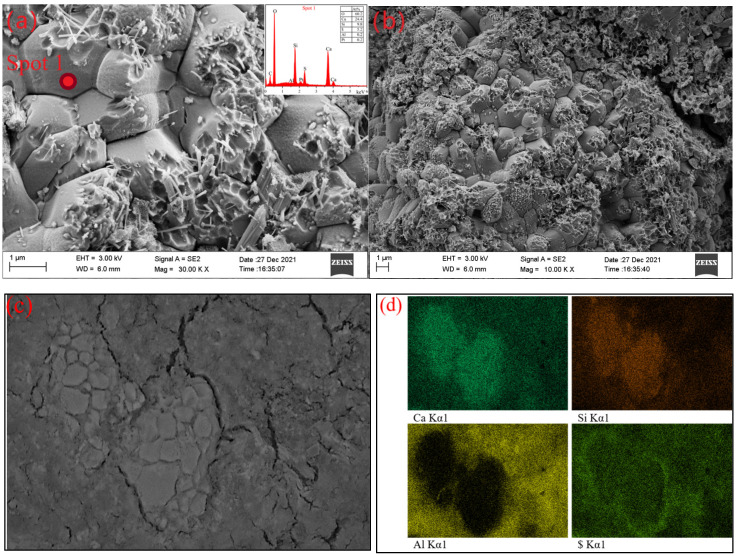
(**a**,**b**) SEM image of ternesite at 7 d of hydration (on fractured surfaces); (**c**) the selected BSE area of ternesite at 7 d (on polished sections); (**d**) EDS mapping (BSE area).

SEM images (Figure 9a,b) show the morphology of ternesite at 7 d of hydration. The etch pits are present in abundance on the surface of ternesite, and there are abundant needle ettringite in the depressions. The proper explanation is with the reaction between AH_3_ and ternesite. Ternesite gradually loses part of its structure, which leads to nucleation of small pits on the surface. Needle ettringite on the surface can be regarded as the hydration product of ternesite in an Al-rich pore solution [13,28]. The selected BSE area of ternesite is shown in Figure 9c, and elemental maps are depicted in Figure 9d. It can be observed that Ca and Si are enriched in the ternesite-rich zone, while Al is basically outside the ternesite rich zone. S is distributed in all areas, but it is extremely abundant at the ternesite boundary. This may be caused by the accumulation of ettringite on the surface of ternesite.

### 3.6. Proposed Hydration Mechanisms of Ternesite

**Figure 10:**
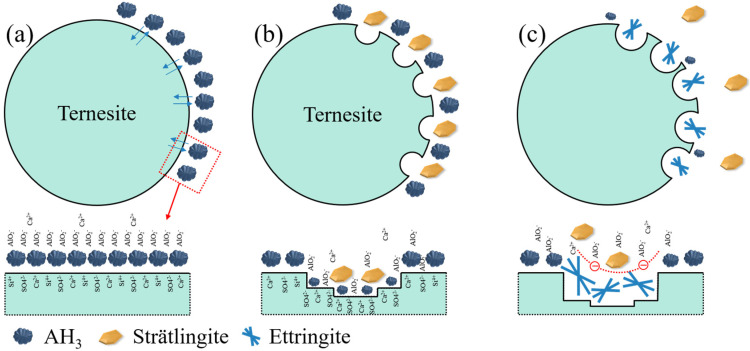
Hydration scheme of ternesite; (**a**) the AH_3_ touches the surface of ternesite; (**b**) ternesite reacts with AH_3_ to form strätlingite; (**c**) the remaining AH_3_ reacts with CaSO_4_ to form ettringite.

Based on the analysis above, it can be stated that the reaction between ternesite and AH_3_ mainly occurs on the surface of ternesite. The prerequisite of reaction is that there is enough AH_3_ around the ternesite. The hydration process on the surface of ternesite follows the steps shown in Figure 10. Firstly, ternesite reacts with AH_3_ in direct contact to form strätlingite and CaSO_4_ by means of ion exchange (Equation (5)). Meanwhile, pits will be formed on the surface of ternesite, and the concentration of AH_3_ will also decrease. The CaSO_4_ formed by hydration of ternesite will continue to react with AH_3_ near the pits to form ettringite, which also requires the participation of Ca^2+^ diffused from other areas. The needle ettringite accumulates on the surface of ternesite and inhibits ion migration, thus restricting further hydration of ternesite.

### 3.7. Mercury Intrusion Porosimetry

**Figure 11:**
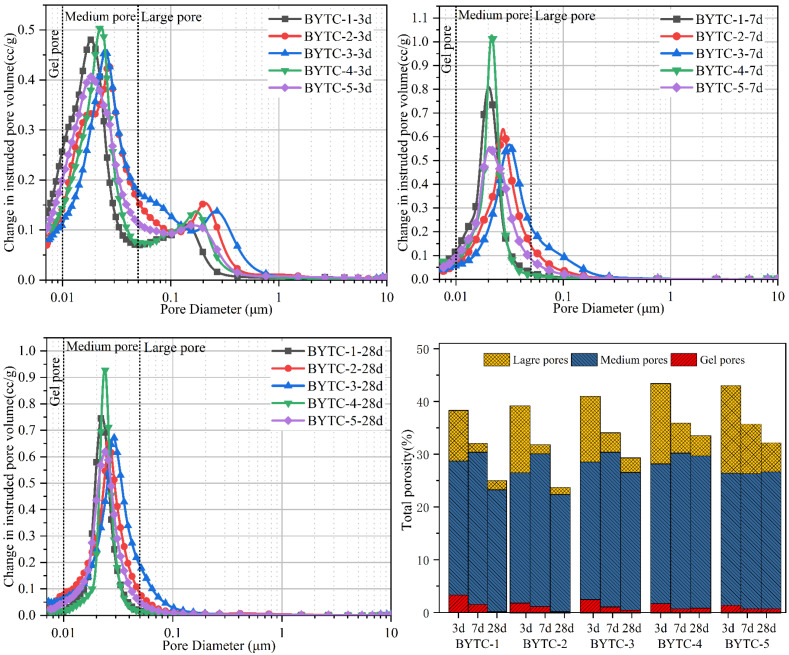
Porosity distribution of blends after 3 d, 7 d and 28 d of hydration. Note: the *y*-axis scale is different.

Figure 11 shows the porosity distribution of BYTC-2 hydration at 3 d and 28 d. According to the effect of different pore sizes on cement performance and hydration, the pore size can be divided into large pores (>0.5 μm), medium pores (0.01–0.5 μm) and gel pores (<0.01 μm). The pore volume of BYT cement after hydration is higher on day 3 than on the other days, and the pore has double peaks or a wide peak, which indicates that the pore is widely distributed. After hydration for 7 d, the pore distribution of the paste is concentrated in the medium pores, and the number of large pores and gel pores decreases. The growth of ettringite transforms the large pores into medium pores, making the cement paste denser. On 28 d, a large number of pores larger than 0.1 microns in diameter were filled by hydration products, which reduced the pore volume, and the pores larger than 0.1 microns in the BYT cement samples basically disappeared. The appearance of pore peaks is related not only to the concentrated distribution of pore sizes but also to the strength of cement paste. The pore volume of the BYTC-2 sample is basically lower than that of the other samples, which is consistent with its good strength.

### 3.8. Compressive Strength

**Figure 12:**
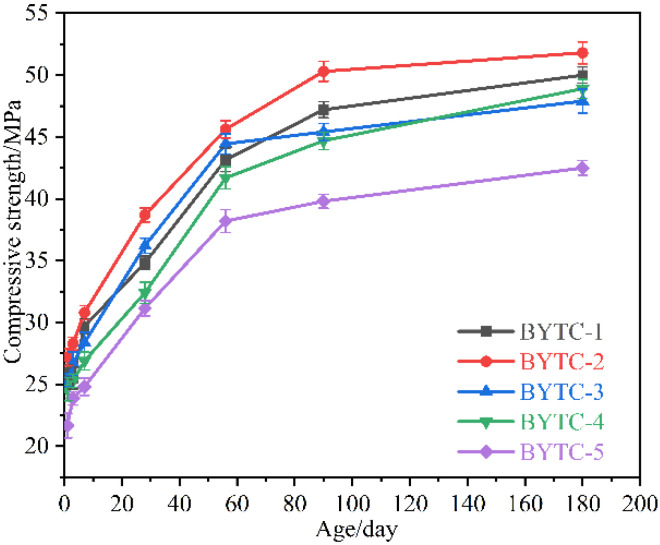
Compressive strength of BYT cement, prepared at *w*/*c* = 0.5.

**Figure 13:**
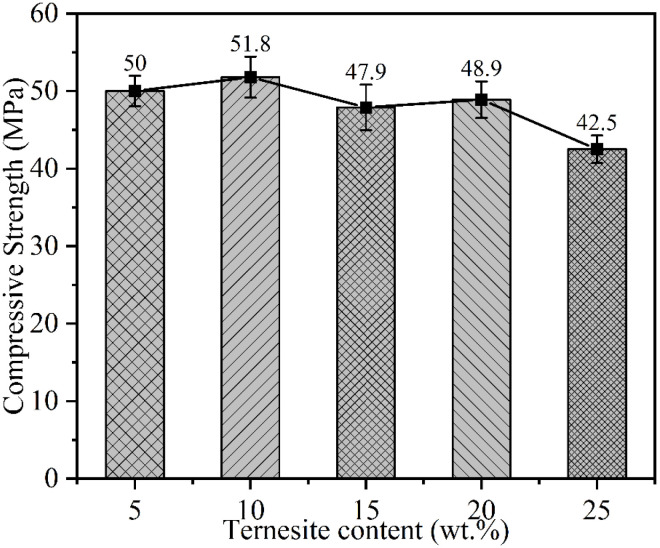
The compressive strength (180 d) of BYT cement relation with the ternesite content in the clinker.

Figure 12 shows the compressive strength development of BYT cement. The compressive strength of the BYT sample within 56 d of hydration age is greatly affected by the content of ternesite. The higher the content of ternesite, the weaker the early strength. After hydration for more than 56 d, the strength still grows slowly, and there is no strength decrease phenomenon. This can be explained by the continued hydration of ternesite and belite in the system. It can be seen from the figure that with the extension of hydration time, the compressive strength of each sample presents a slightly upward trend. The highest compressive strength for each age is observed with the BYTC-2 sample, of which compressive strengths of 90 d and 180 d reach 51 MPa and 52 MPa, respectively. Figure 13 shows the compressive strength of the BYT cement relation with the ternesite content in the clinker after 180 d of hydration. As the content of ternesite samples reached 10%, the compressive strength performance was better than that of the other samples after 180 d of hydration.

## 4. Conclusions

In this paper, the hydration process and hydration products of BYT cement clinker and the compressive strength of BYT cement were studied. The synergistic effect among belite, ye′elimite and ternesite was discussed, and the following conclusions were drawn:

(1) The presence of ternesite in cement delays the early hydration of ye′elimite, resulting in a low rate of early hydration. The higher the content of ternesite in the system, the later the second hydrating exothermic peak.
(2) Ternesite showed good hydration activity in the hydration process of BYT cement. The presence of ternesite promotes belite hydration activity. With increasing hydration age, the content of ettringite increased, and the crystallinity improved, so the intensity of the diffraction peak of ettringite gradually strengthened. AH_3_ precipitated in an amorphous form and was not detected by XRD. The hydration of ternesite mainly occurs on the surface. The presence of ettringite on the surface of ternesite may hinder further hydration of ternesite.
(3) With the increase in hydration time, the pore volume of cement paste decreased in each sample, which promoted and improved the strength of the cement sample. The compressive strength of the BYT cement samples did not reverse when the hydration age exceeded 56 d but still grew slowly, which was due to the involvement of ternesite in the system during hydration. The strength of BYTC-2 (containing 10% ternesite in the prepared clinker) exceeds that of other cements at all ages.

## Figures and Tables

**Table 1:** Design phase composition of clinker and amount of raw materials (wt.%).

Sample No.	Raw Materials Dosage				Design Phase Composition		
	CaCO_3_	SiO_2_	Al_2_O_3_	CaSO_4_·H_2_O	C_2_S	C_4_A_3_$	C_5_S_2_$
BYT-1	60.24	13.33	16.10	10.33	50	45	5
BYT-2	57.30	11.86	18.09	12.75	40	50	10
BYT-3	58.18	12.69	16.18	12.95	40	45	15
BYT-4	51.21	8.83	22.20	17.76	20	60	20
BYT-5	56.09	12.05	16.26	15.60	30	45	25

**Table 2:** The mixture of gypsum and clinker (wt.%).

Sample No.	BYTC-1	BYTC-2	BYTC-3	BYTC-4	BYTC-5
Clinker	79.79	78.16	80.12	74.85	79.86
Gypsum	20.21	21.84	19.88	25.15	20.14

**Table 3:** The chemical composition and μm value of BYT clinkers.

Oxide (wt.%)	BYT-1	BYT-2	BYT-3	BYT-4	BYT-5
CaO	52.15	50.48	51.58	46.87	50.68
SiO_2_	18.69	16.58	17.98	12.28	16.78
Al_2_O_3_	22.52	24.88	22.32	29.85	22.52
SO_3_	6.64	8.01	8.03	11.07	9.99
μ_m_ (cm^2^/g)	81.41	79.88	80.95	76.81	80.50

**Table 4:** ICSD collection codes and references for all phases used in the Rietveld quantitative phase analysis.

Phase	ICSD Code	Ref.	Phase	ICSD Code	Ref.
C_2_S	81096	[34]	CaO	79784	[35]
C_5_S_2_$	85123	[36]	CaSO_4_	1956	[37]
SiO_2_	174	[38]	CaSO_4_·2H_2_O	27876	[39]
C_4_A_3_$-o	80361	[40]	Ettringite	16045	[41]
C_4_A_3_$-c	9560	[42]			

**Table 5:** Phase composition measured by XRD-Rietveld refinement and Rwp values for all clinkers.

Phase (wt.%)	BYT-1	BYT-2	BYT-3	BYT-4	BYT-5
C_2_S	49.1	39.8	39.8	19.8	29.5
C_4_A_3_$-o	32.1	38.7	30.5	43.4	34.7
C_4_A_3_$-c	13.2	11.3	13.9	16.7	10.4
C_4_A_3_$-sum	45.3	50.0	44.4	60.1	45.1
C_5_S_2_$	5.1	9.9	14.9	19.8	24.8
CaSO_4_	0.0	0.2	0.6	0.0	0.0
SiO_2_	0.5	0.0	0.2	0.2	0.6
Rwp	8.64	9.32	8.56	9.58	8.96

## Data Availability

Not applicable.

## References

[1] Naqi A., Jang J.G. (2019). Recent Progress in Green Cement Technology Utilizing Low-Carbon Emission Fuels and Raw Materials: A Review. Sustainability.

[2] Gartner E.M., Quillin K. Low-CO_2_ cements based on calcium sulfoaluminates. Proceedings of the International Conference on Sustainability in the Cement and Concrete Industry.

[3] Hanein T., Galvez-Martos J.-L., Bannerman M. (2018). Carbon footprint of calcium sulfoaluminate clinker production. J. Clean. Prod..

[4] Ioannou S., Reig L., Paine K., Quillin K. (2014). Properties of a ternary calcium sulfoaluminate–calcium sulfate–fly ash cement. Cem. Concr. Res..

[5] Gartner E. (2004). Industrially interesting approaches to ‘low-CO_2_’ cements. Cem. Concr. Res..

[6] Bullerjahn F., Zajac M., Ben Haha M. (2015). CSA raw mix design: Effect on clinker formation and reactivity. Mater. Struct..

[7] Bertola F., Gastaldi D., Irico S., Paul G., Canonico F. (2020). Behavior of blends of CSA and Portland cements in high chloride environment. Constr. Build. Mater..

[8] Li P., Gao X., Wang K., Tam V.W., Li W. (2020). Hydration mechanism and early frost resistance of calcium sulfoaluminate cement concrete. Constr. Build. Mater..

[9] Chen I.A., Juenger M.C. (2012). Incorporation of coal combustion residuals into calcium sulfoaluminate-belite cement clinkers. Cem. Concr. Compos..

[10] Chen I.A., Hargis C.W., Juenger M.C. (2012). Understanding expansion in calcium sulfoaluminate–belite cements. Cem. Concr. Res..

[11] Bullerjahn F., Schmitt D., Ben Haha M. (2014). Effect of raw mix design and of clinkering process on the formation and mineralogical composition of (ternesite) belite calcium sulphoaluminate ferrite clinker. Cem. Concr. Res..

[12] Böhme N., Hauke K., Neuroth M., Geisler T. (2020). In Situ Hyperspectral Raman Imaging of Ternesite Formation and Decomposition at High Temperatures. Minerals.

[13] Bullerjahn F., Haha M.B. (2013). Belite-Calciumsulfoaluminate-Ternesite (BCT)—A new low carbon clinker technology. Cem. Int..

[14] Bullerjahn F., Boehm-Courjault E., Zajac M., Ben Haha M., Scrivener K. (2019). Hydration reactions and stages of clinker composed mainly of stoichiometric ye’elimite. Cem. Concr. Res..

[15] Winnefeld F., Lothenbach B. (2010). Hydration of calcium sulfoaluminate cements—Experimental findings and thermodynamic modelling. Cem. Concr. Res..

[16] Jen G., Skalamprinos S., Whittaker M., Galan I., Imbabi M.S., Glasser F.P. (2017). The impact of intrinsic anhydrite in an experimental calcium sulfoaluminate cement from a novel, carbon-minimized production process. Mater. Struct..

[17] Winnefeld F., Barlag S. (2009). Influence of calcium sulfate and calcium hydroxide on the hydration of calcium sulfoaluminate clinker. ZKG Int..

[18] Beltagui H., Jen G., Whittaker M., Imbabi M.S. (2017). The influence of variable gypsum and water content on the strength and hydration of a belite-calcium sulphoaluminate cement. Adv. Appl. Ceram..

[19] Christensen A.N., Jensen T.R., Hanson J.C. (2004). Formation of ettringite, Ca_6_Al_2_(SO_4_)_3_(OH)_12_·26H_2_O, AFt, and monosulfate, Ca_4_Al_2_O_6_(SO_4_)·14H_2_O, AFm-14, in hydrothermal hydration of Portland cement and of calcium aluminum oxide—Calcium sulfate dihydrate mixtures studied by in situ synchrotron X-ray powder diffraction. J. Solid State Chem..

[20] Winnefeld F., Lothenbach B. (2016). Phase equilibria in the system Ca_4_Al_6_O_12_SO_4_–Ca_2_SiO_4_–CaSO_4_–H_2_O referring to the hydration of calcium sulfoaluminate cements. RILEM Tech. Lett..

[21] Berger S., Coumes C.C.D., Le Bescop P., Damidot D. (2011). Influence of a thermal cycle at early age on the hydration of calcium sulphoaluminate cements with variable gypsum contents. Cem. Concr. Res..

[22] Kaprálik I., Hanic F. (1989). Phase relations in the subsystem C_4_A_3_S-CSH_2_-CH-H_2_O of the system CaO-Al_2_O_3_-CS-H_2_O referred to hydration of sulphoaluminate cement. Cem. Concr. Res..

[23] Glasser F., Zhang L. (2001). High-performance cement matrices based on calcium sulfoaluminate–belite compositions. Cem. Concr. Res..

[24] Shirani S., Cuesta A., Morales-Cantero A., De la Torre A.G., Olbinado M.P., Aranda M.A. (2021). Influence of curing temperature on belite cement hydration: A comparative study with Portland cement. Cem. Concr. Res..

[25] Cook R., Ma H., Okoronkwo M., Sant G., Kumar A. (2020). Influence of water activity on belite (β-C2S) hydration. J. Am. Ceram. Soc..

[26] Andac M., Glasser F.P. (1999). Pore solution composition of calcium sulfoaluminate cement. Adv. Cem. Res..

[27] Juenger M., Winnefeld F., Provis J., Ideker J. (2011). Advances in alternative cementitious binders. Cem. Concr. Res..

[28] Haha M.B., Bullerjahn F., Maciej Z. On the reactivity of ternesite. Proceedings of the 14th International Congress on the Chemistry of Cement.

[29] Montes M., Pato E., Carmona-Quiroga P., Blanco-Varela M. (2018). Can calcium aluminates activate ternesite hydration?. Cem. Concr. Res..

[30] Carmona-Quiroga P., Montes M., Pato E., Fernández-Jiménez A., Blanco-Varela M. (2020). Study on the activation of ternesite in CaO·Al2O3 and 12CaO·7Al2O3 blends with gypsum for the development of low-CO_2_ binders. J. Clean. Prod..

[31] Shen Y., Wang P., Chen X., Zhang W., Qian J. (2020). Synthesis, characterisation and hydration of ternesite. Constr. Build. Mater..

[32] Zhang L. (2000). Microstructure and Performance of Calcium Sulfoaluminate Cements. Ph.D. Dissertation.

[33] Li X., Snellings R., Scrivener K.L. (2019). Quantification of amorphous siliceous fly ash in hydrated blended cement pastes by X-ray powder diffraction. J. Appl. Crystallogr..

[34] Gmumme W., Hill R.J., Bushnell-Wye G. (1995). Rietveld crystal structure refinements, crystal chemistry and calculated powder diffraction data for the polymorphs of dicalcium silicate and related phases. Neues Jahrb. Fuer Mineral.-Abh..

[35] Huang Q., Soubeyroux J., Chmaissem O., Sora I., Santoro A., Cava R., Krajewski J., Peck W. (1994). Neutron Powder Diffraction Study of the Crystal Structures of Sr2RuO4 and Sr2IrO4 at Room Temperature and at 10 K. J. Solid State Chem..

[36] Irran E., Tillmanns E., Hentschel G. (1997). Ternesite, Ca_5_(SiO_4_)_2_SO_4_, a new mineral from the Ettringer Bellerberg/Eifel, Germany. Miner. Pet..

[37] Morikawa H., Minato I., Tomita T., Iwai S. (1975). Anhydrite: A refinement. Acta Crystallogr. Sect. B Struct. Crystallogr. Cryst. Chem..

[38] Le Page Y., Donnay G. (1976). Refinement of the crystal structure of low-quartz. Acta Crystallogr. Sect. B Struct. Crystallogr. Cryst. Chem..

[39] Abriel W., Reisdorf K., Pannetier J. (1990). Dehydration reactions of gypsum: A neutron and X-ray diffraction study. J. Solid State Chem..

[40] Calos N.J., Kennard C.H., Whittaker A.K., Davis R. (1995). Structure of calcium aluminate sulfate Ca_4_Al_6_O_16_S. J. Solid State Chem..

[41] Moore A.E., Taylor H.F.W. (1970). Crystal structure of ettringite. Acta Crystallogr. Sect. B Struct. Crystallogr. Cryst. Chem..

[42] Saalfeld H., Depmeier W. (1972). Silicon-Free Compounds with Sodalite Structure. Cryst. Res. Technol..

[43] Bullerjahn F., Zajac M., Skocek J., Ben Haha M. (2019). The role of boron during the early hydration of belite ye’elimite ferrite cements. Constr. Build. Mater..

[44] Telesca A., Marroccoli M., Coppola L., Coffetti D., Candamano S. (2021). Tartaric acid effects on hydration development and physico-mechanical properties of blended calcium sulphoaluminate cements. Cem. Concr. Compos..

[45] Song F., Yu Z., Yang F., Lu Y., Liu Y. (2015). Microstructure of amorphous aluminum hydroxide in belite-calcium sulfoaluminate cement. Cem. Concr. Res..

[46] Padilla-Encinas P., Palomo A., Blanco-Varela M., Fernández-Jiménez A. (2020). Calcium sulfoaluminate clinker hydration at different alkali concentrations. Cem. Concr. Res..

[47] Zhang Y., Chang J., Ji J. (2018). AH3 phase in the hydration product system of AFt-AFm-AH3 in calcium sulfoaluminate cements: A microstructural study. Constr. Build. Mater..

